# Glioma-intrinsic SLC1A3 hijacks the vascular niche to establish an immunosuppressive microenvironment

**DOI:** 10.3389/fimmu.2026.1824726

**Published:** 2026-04-23

**Authors:** Hao Lin, Chaxian Liu, Xi Chen, Yingbo Zhao, Yi Lyu, Bilong Zhang, Haikun Song, Xiaomin Fan, Shasha Li, Ziqian He, Hui Yang, Ying Mao

**Affiliations:** 1 Department of Neurosurgery, Huashan Hospital, Fudan University, Shanghai, China; 2 Institute for Translational Brain Research, Shanghai Medical College, Fudan University, Shanghai, China; 3 National Center for Neurological Disorders, Huashan Hospital, Shanghai Medical College, Fudan University, Shanghai, China; 4 Shanghai Key Laboratory of Brain Function Restoration and Neural Regeneration, Shanghai Clinical Medical Center of Neurosurgery, Neurosurgical Institute of Fudan University, Huashan Hospital, Shanghai Medical College, Fudan University, Shanghai, China; 5 State Key Laboratory of Medical Neurobiology and Ministry of Education (MOE) Frontiers Center for Brain Science, Shanghai Medical College, Fudan University, Shanghai, China; 6 Department of Biostatistics, Mailman School of Public Health, Columbia University, New York, NY, United States

**Keywords:** alternative splicing, endothelial cells, foundation models (Geneformer), glioblastoma, glioma-initiating cells, immune evasion, single-cell transcriptomics, tumor microenvironment

## Abstract

**Introduction:**

Glioblastoma (GBM) is a highly lethal malignancy driven by glioma-initiating cells (GICs). While GICs are known to profoundly remodel tumor microenvironment (TME) to promote progression and immune evasion within the vascular niche, the specific transcriptomic reprogramming and alternative splicing events driving their evolution from neural stem cells (NSCs), and how these intrinsic cellular state changes dictate multi-cellular immunosuppressive networks and checkpoints, remain poorly understood. Unraveling these complex tumor-vascular-immune interactions is critical for identifying novel vulnerabilities and developing effective immunotherapies.

**Methods:**

To decode the GICs’ evolutionary trajectory, we integrated RNA-seq and alternative splicing analysis of NSCs and patient-derived GIC cohorts. The malignant progression was mapped using scRNA-seq pseudotime analysis, and key targets were validated across clinical TCGA cohorts. Furthermore, we employed the large-scale single-cell foundation model, Geneformer, to perform in silico genetic perturbations, integrating it with interactome inference to decipher TME communication. Finally, the proposed tumor-endothelial-T cell multi-cellular axis was functionally validated utilizing *in vitro* tumor-HUVEC co-culture systems, qPCR, and FACS-based T cell activation (NFAT-Jurkat) assays.

**Results:**

Our multi-omics re-analysis identified extensive alternative splicing and transcriptional reprogramming during GICs evolution, pinpointing SLC1A3 as a core gene significantly upregulated along the malignant pseudotime trajectory and strongly correlated with poor clinical prognosis in GBM. AI-driven in silico virtual knockout utilizing Geneformer revealed that SLC1A3 acts as a master regulator of tumor network stability. Interactome analysis demonstrated that SLC1A3^hi^ tumor cells exhibit intensive communication with endothelial cells via specific ligand-receptor axes (e.g., TNC-ITGB1, PTN-SDC3). *In vitro* assays confirmed that endothelial cells were educated by SLC1A3^hi^ tumor cells that undergo malignant transition, drastically upregulating immune-suppressive factors, including *CD274*, *TGFB1*, *IL10*, and *IDO1*. Crucially, tumor-specific knockdown of *SLC1A3* dismantled this vascular-immune suppressive niche, significantly restoring T cell activation in a multicellular co-culture model.

**Discussion:**

Our findings establish SLC1A3 not merely as an intrinsic driver of glioma development, but as a critical upstream node orchestrating a cascading tumor-endothelial-T cell immunosuppressive axis. By leveraging AI-based foundation models alongside robust biological validation, we uncovered a novel mechanism of vascular-mediated immune evasion, highlighting SLC1A3 as a highly promising therapeutic target to reprogram the glioblastoma microenvironment and restore anti-tumor immunity.

## 1 Introduction

Glioblastoma (GBM) is the most prevalent and aggressive primary malignant brain tumor in adults, characterized by profound cellular heterogeneity, relentless invasiveness, and a dismal median survival of approximately 15 months despite maximal multimodal therapy (1–3). The inevitable recurrence and therapeutic resistance of GBM are largely driven by glioma-initiating cells (GICs), also known as glioma stem cells (GSCs) (4), a highly plastic subpopulation that hijacks neurodevelopmental programs and originates from the malignant transformation of neural stem cells (NSCs) (5, 6). Recent advancements in single-cell and spatial transcriptomics have revealed that GICs do not exist in isolation; rather, they orchestrate a highly immunosuppressive tumor microenvironment (TME) by extensively remodeling the perivascular niche and paralyzing infiltrating immune cells (7–9). While the phenotypic endpoints of GICs-mediated immune evasion are well-documented, the dynamic transcriptomic reprogramming and complex regulatory events that dictate the evolutionary trajectory from normal NSCs to malignant GICs remain poorly understood (10, 11). Crucially, how these intrinsic evolutionary alterations within GICs cascade to systematically dictate multicellular crosstalk, specifically the instruction of endothelial cells to establish a T cell-exclusive vascular niche, represents a critical void in contemporary neuro-oncology (12, 13).

Alternative splicing (AS) has emerged as a fundamental post-transcriptional mechanism governing transcriptomic diversity, playing a pivotal role in normal neurogenesis and being frequently hijacked during oncogenesis to drive tumor plasticity, metabolic adaptation, and immune evasion (14–16). However, the systematic AS landscape underlying the NSCs-to-GICs transition remains largely unexplored. Furthermore, traditional differential gene expression and trajectory analysis are fundamentally limited by their inability to decipher the global network vulnerabilities underlying these complex multicellular interactions (17). Recently, the advent of large-scale single-cell foundation models based on transformer architectures (e.g., Geneformer, scGPT) has revolutionized computational biology (18–20). Pre-trained on tens of millions of cellular transcriptomes, these AI models possess the unprecedented capability to comprehend context-aware gene network dynamics and perform in silico genetic perturbations to predict cell state shifts (21, 22). Despite this paradigm shift, leveraging AI-driven foundation models to decode the specific molecular drivers of glioma evolution and their subsequent impact on the vascular-immune axis has not yet been achieved.

In this study, we employed an integrative multi-omics framework, combining bulk RNA-seq AS profiling with single-cell pseudotime trajectory analysis, to decode the evolutionary transition from NSCs to patient-derived GICs. This comprehensive approach identified massive AS events and pinpointed SLC1A3 as a core malignant driver significantly upregulated along the glioma evolutionary trajectory, correlating strongly with poor clinical prognosis. Strikingly, by utilizing the Geneformer foundation model (316M parameters) for in silico virtual knockout, we unveiled SLC1A3 as a master regulator safeguarding the transcriptomic network stability of glioma cells. Integrated with multicellular interactome inference, we uncovered that SLC1A3^hi^ tumor cells construct a tri-directional immunosuppressive network: tumor cells secrete core ligands (e.g., TNC, PTN) to activate the endothelial cell niche, which subsequently upregulates critical immunosuppressive factors (e.g., *CD274*, *TGFB1*, *IL10*, and *IDO1*) to restrict T cell activation. Finally, utilizing *in vitro* tumor-HUVEC co-culture systems and FACS-based NFAT-Jurkat reporter assays, we functionally validated that targeted ablation of SLC1A3 dismantles this vascular-immune suppressive cascade, effectively restoring T cell activation. Collectively, our study unveils the AI-predicted and experimentally validated role of SLC1A3 in orchestrating a malignant tumor-endothelial-T cell axis, offering a novel therapeutic vulnerability for GBM microenvironment reprogramming.

## 2 Results

### 2.1 Transcriptome signatures reveal unique intrinsic changes during glioma development

**Figure 1:**
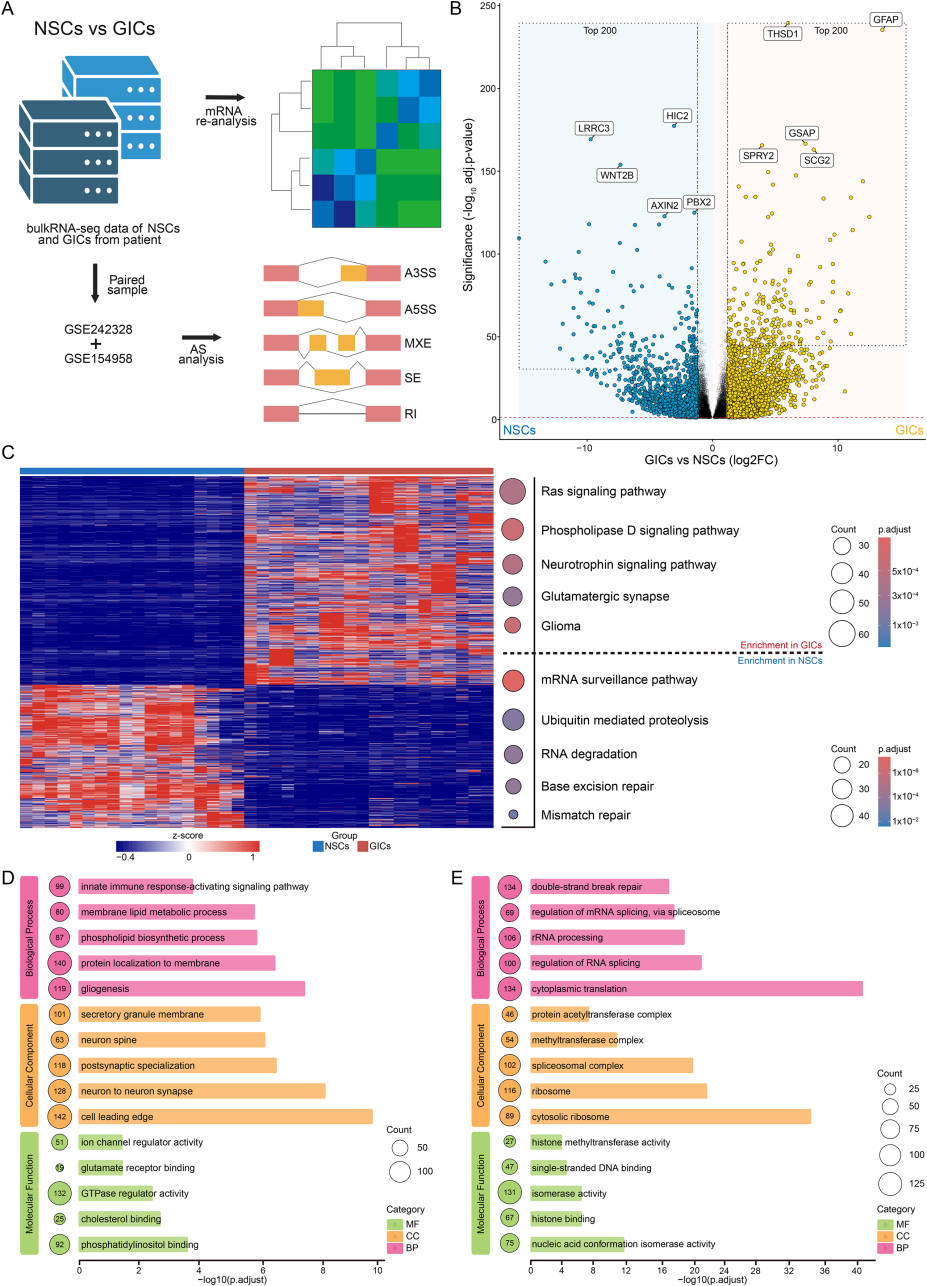
Transcriptomic signatures reveal unique intrinsic changes during glioma development. **(A)** The workflow of analysis of RNA-seq data. **(B)** The volcano plot of differentially expressed genes between GICs and NSCs, and the five most significantly up-regulated/down-regulated genes are shown. **(C)** The heatmap of DEGs between GICs and NSCs, with the KEGG enrichment results of shared upregulated genes in GICs, and the enrichment results of shared upregulated genes in NSCs. **(D)** GO enrichment results of shared upregulated genes in the two datasets in GICs. **(E)** GO enrichment results of shared upregulated genes in the two datasets in NSCs.

To investigate the transcriptomic alterations driving gliomagenesis, we leveraged two independent IDH1 wild-type GBM RNA-seq datasets (GSE242328 and GSE154958) (23, 24) comprising patient-derived GICs and matched NSCs (**Figure 1A**). Differential expression analysis of the raw transcriptomic data revealed substantial reprogramming, as visualized by volcano plots that highlighted key differentially expressed genes (DEGs) between the GICs and NSC populations (**Figure 1B**). Notably, the expression of the canonical glial marker GFAP was markedly upregulated in the neoplastic compartment relative to NSCs, consistent with glioma pathobiology. Hierarchical clustering of these DEGs, represented by heatmaps (**Figure 1C**), further delineated the highly divergent transcriptional landscapes distinguishing GICs from NSCs. Subsequently, to distill core oncogenic signatures, we intersected the datasets and performed Kyoto Encyclopedia of Genes and Genomes (KEGG) (**Figure 1C**, right) and Gene Ontology (GO) (**Figures 1D, E**) enrichment analysis on the consensus upregulated genes within either GICs or NSCs. Pathway enrichment revealed that, during glioma progression, GICs exhibited a robust overrepresentation of immune reprogramming, functional networks linked to synaptic transmission and oncogenesis relative to NSCs, concomitantly with profound perturbations in membrane-associated signatures. Conversely, NSCs retained significant enrichment for nucleotide repair pathways compared to GICs, implicating the abrogation of DNA maintenance and ensuing genomic instability as pivotal drivers in the pathogenesis of glioma, although great changes in AS events.

Collectively, our initial transcriptomic profiling delineates a distinct transcriptional reprogramming landscape during early gliomagenesis. This process is characterized by prominent immune response, neuro-synaptic crosstalk, putative remodeling of membrane architecture, and a profound dysregulation of transcriptome homeostasis within the emerging neoplastic compartment.

### 2.2 Differential alternative splicing exhibits a unique transcriptional landscape in GICs

**Figure 2:**
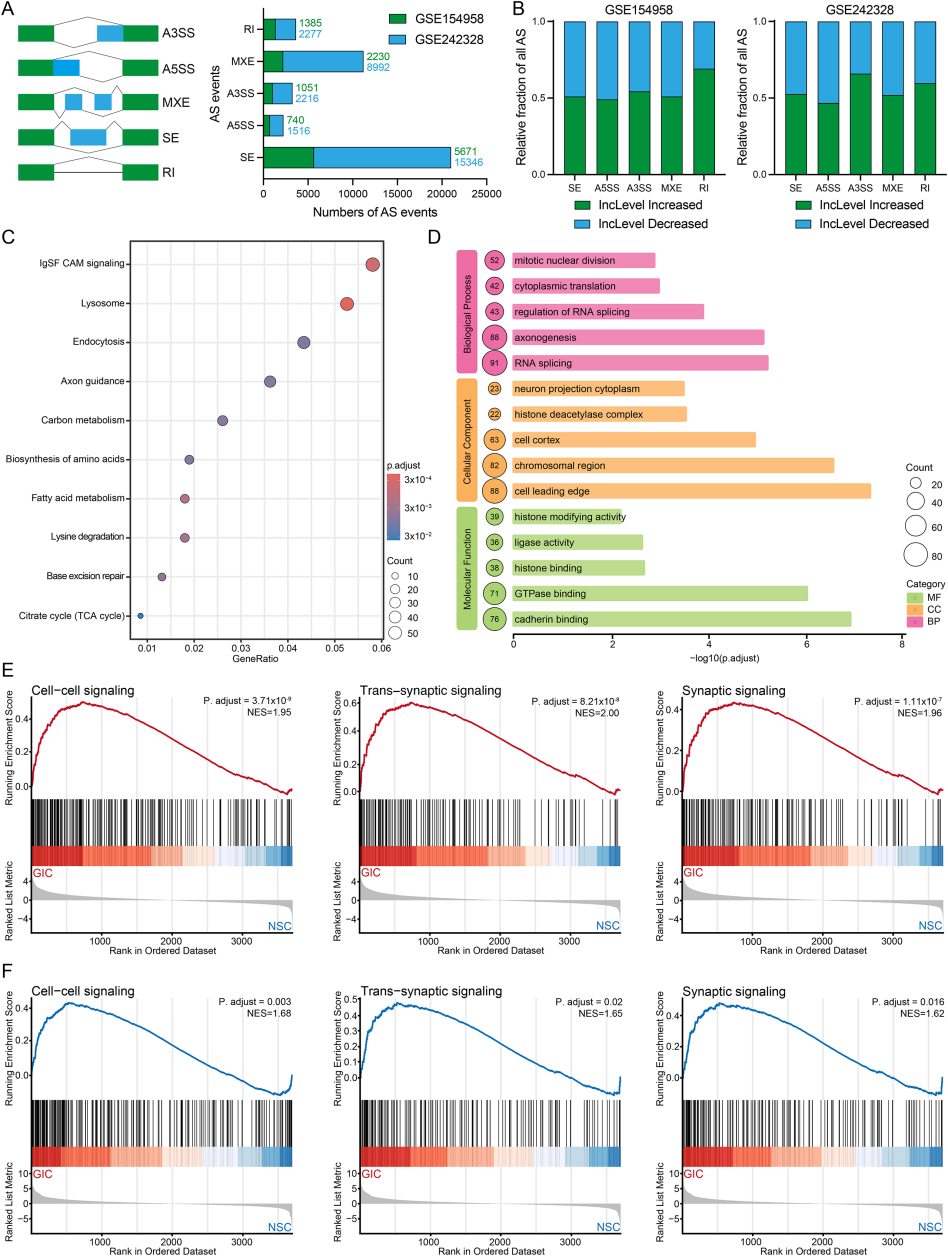
Differential analysis of alternative splicing during glioma development. **(A)** The overview of all differential AS events in GICs and NSCs. **(B)** The overview of all differential AS events proportion in GICs and NSCs. **(C)** KEGG enrichment results of upregulated differential AS genes in the two datasets in GICs. **(D)** GO enrichment results of upregulated differential AS genes shared in the two datasets in GICs. **(E)** The GSEA enrichment results of neural development pathways in GICs in GSE242328. **(F)** The GSEA enrichment results of neural development pathways in GICs in GSE154958.

Beyond macroscopic transcriptional shifts, AS represents a sophisticated post-transcriptional RNA processing mechanism that is strictly required for proper neurodevelopment; thus, we next investigated whether the dysregulation of AS dynamics contributes to gliomagenesis. We then utilized rMATS (25) to perform AS analysis on RNA-seq data from NSCs and GICs. Our results revealed many AS events between NSCs and GICs, with skipped exon (SE) being the most prevalent event type (**Figure 2A**). Notably, the differential AS events between two independent datasets remained broadly consistent (**Figure 2B**). To assess potential event preferences among these AS genes, we selected and integrated co-upregulated AS genes shared between two independent datasets, followed by downstream KEGG and GO analysis (**Figures 2C, D**). Our findings indicate that the upregulated AS genes in GICs primarily involve invasion, RNA splicing, histone modification, neuron development, and nucleoplasmic transport. Prompted by the striking concordance of neurodevelopmental and regulatory pathways enriched across both global transcriptional and AS profiles in GICs, we performed gene set enrichment analysis (GSEA) to further substantiate these findings. Remarkably, across both independent datasets (**Figures 2E, F**), we observed robust and uniform enrichment of networks governing cell-cell communication and synaptic transmission in the tumor compartment. These results demonstrate that, relative to matched NSCs, GICs acquire a markedly heightened neural activity signature.

In summary, aberrant AS events during gliomagenesis are predominantly enriched in networks governing neurodevelopment, synaptic transmission, epigenetic remodeling, and tumor invasion. This suggests that AS reprogramming acts as a crucial post-transcriptional driver in orchestrating the heightened neural activity signature and malignant phenotypes of GICs. Targeting these splicing-regulated neural and intercellular communication pathways may offer a promising therapeutic strategy to uncouple the neuro-oncological crosstalk and halt glioma progression.

### 2.3 Integrated transcriptome data reveal the glioma development trajectory

**Figure 3:**
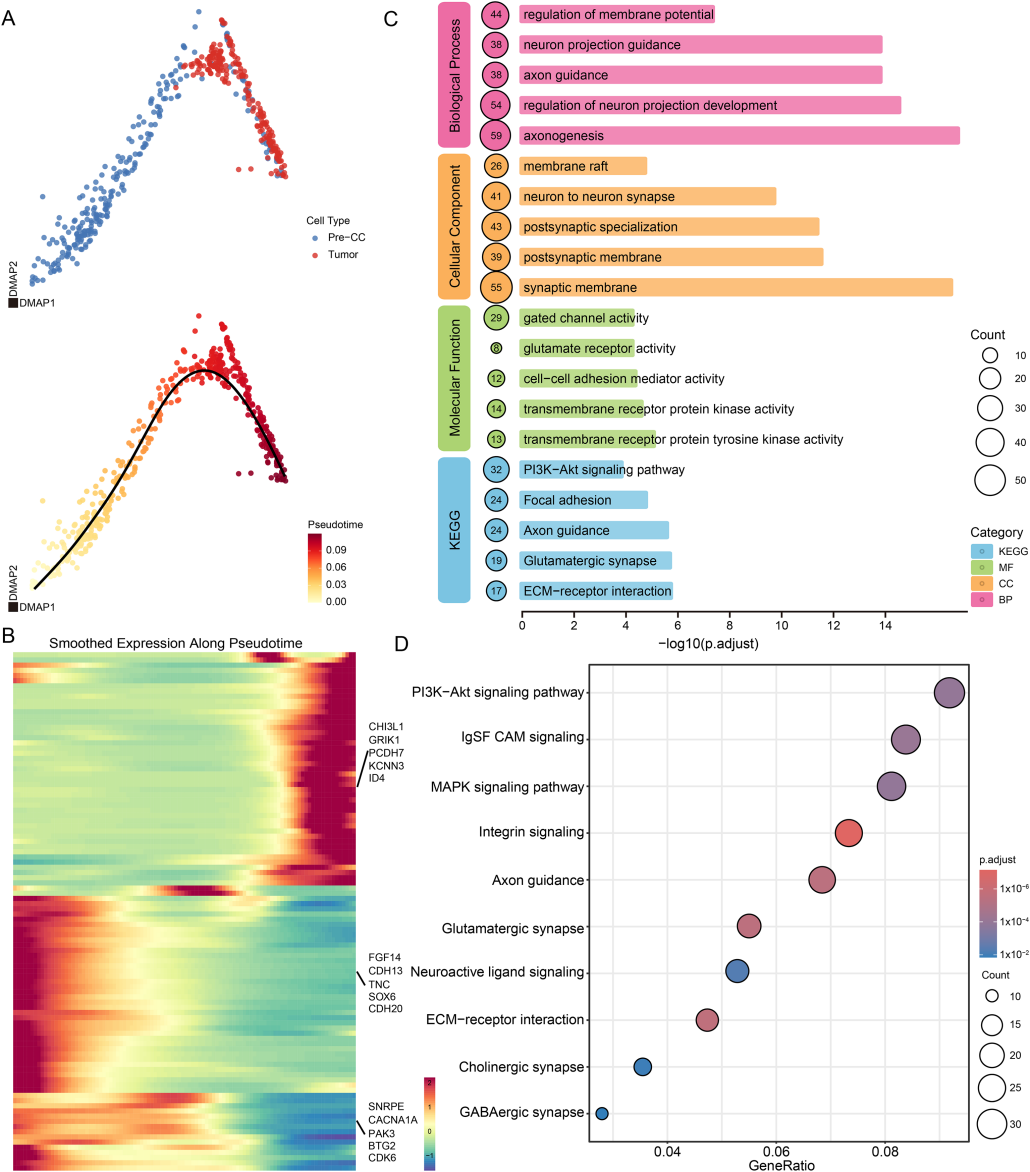
Single-cell pseudo-temporal analysis revealed dynamic changes in signaling pathways during glioma initiation. **(A)** Pseudo-time analysis confirmed the evolutionary trajectory from Pre-CC to tumor. **(B)** Heatmap of time-series differential genes during glioma progression. **(C)** KEGG enrichment results of shared upregulated genes during tumor development. **(D)** GO enrichment results of the shared upregulated genes during tumor development.

To achieve a higher-resolution understanding of the temporal dynamics driving glioma initiation, we leveraged published single-cell RNA sequencing (scRNA-seq) data by Kim et al. (26). We integrated pre-neoplastic cells (Pre-CC) with their matched tumor counterparts to construct a pseudotime evolutionary trajectory (**Figure 3A**). As anticipated, diffusion map-based trajectory inference mapped a continuous developmental continuum, seamlessly transitioning from the Pre-CC state toward fully established neoplastic cells.

To decode the transcriptional waves orchestrating this transition, we modeled dynamic gene expression along pseudotime (**Figure 3B**). During the early phases of gliomagenesis, the transcriptional landscape was dominated by the robust enrichment of canonical developmental regulators and cell cycle modulators, including the CDH, CDK (e.g., CDK6), and SOX (e.g., SOX8) families, highlighting the initial expansion and maintenance of a progenitor-like pool. However, as the trajectory advanced toward the late oncogenic state, this program underwent profound reprogramming. We observed the terminal induction of established malignant glioma markers, notably CHI3L1 and the protocadherin (PCDH) family, dictating an aggressive phenotypic shift.

Crucially, functional enrichment analysis of these temporally dynamic genes unveiled striking microenvironmental dependencies (**Figures 3C, D**). Rather than purely cell-autonomous proliferation, the genes accompanying tumor evolution were overwhelmingly enriched in neurodevelopmental networks (e.g., axonogenesis), profound remodeling of synaptic membranes, and extensive cell-cell interactions (e.g., ECM-receptor interaction and focal adhesion). Furthermore, the prominent emergence of glutamatergic and GABAergic synaptic signatures during this evolution strongly implies that emerging glioma cells actively construct reciprocal connections with the surrounding brain stroma. Collectively, these single-cell temporal dynamics propose that early gliomagenesis is strictly coupled with profound tumor-neural crosstalk and tumor-stroma integration, allowing nascent tumor cells to hijack host neural circuits for progressive malignancy.

### 2.4 Multi-omics analysis revealed SLC1A3 as the key factor in glioma development

**Figure 4:**
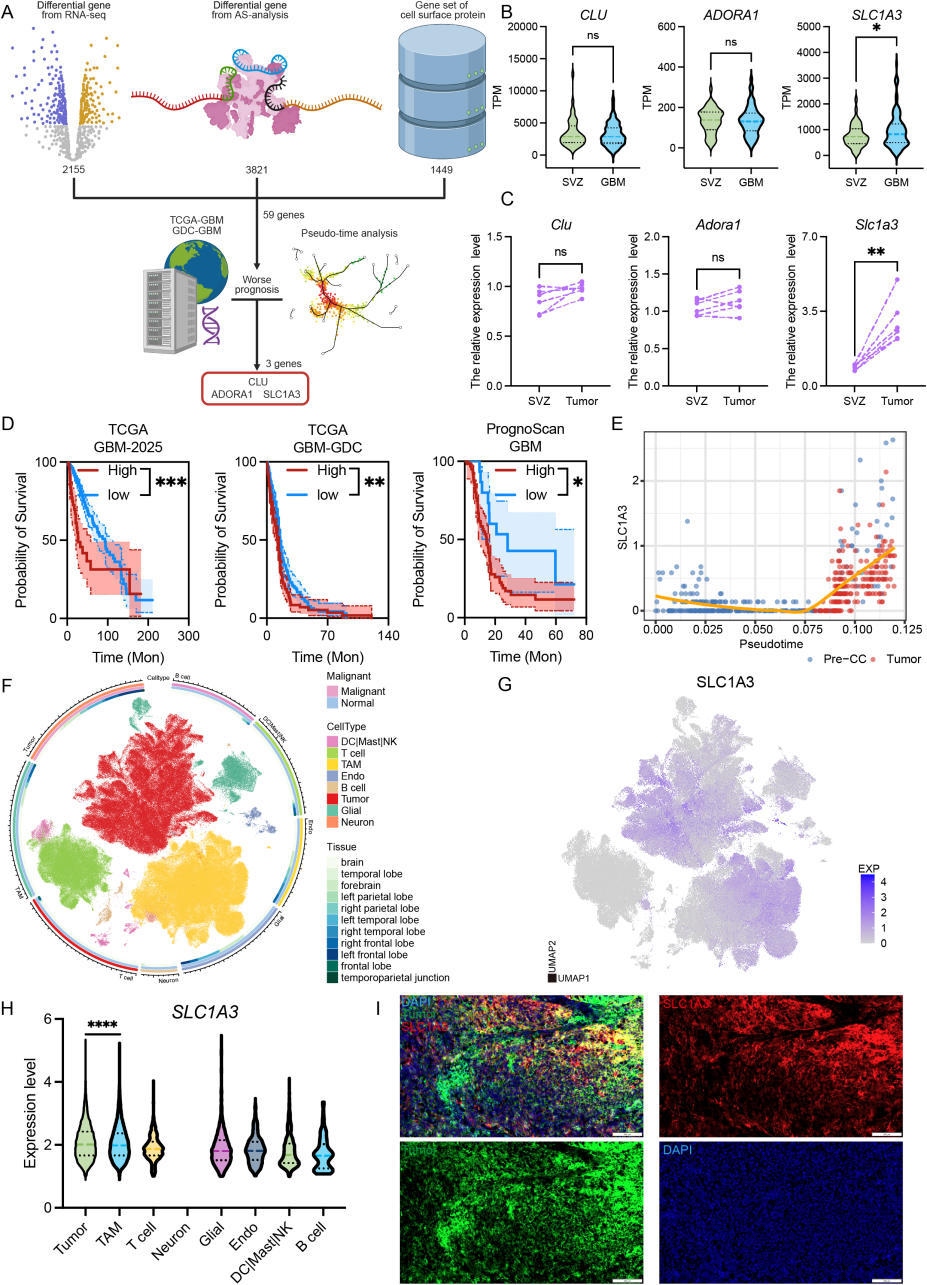
Multi-omics results reveal specific genetic drift events during glioma initiation. **(A)** The workflow of the analysis involves integrating three data sources. **(B)** Comparison of selected gene expression between the SVZ region and the tumor region. **(C)** The quantification of 3 selected genes in the RCAS mouse model in the SVZ and tumor area. The paired Student’s t-test was used for P-value calculation. **(D)** The Kaplan-Meier survival analysis of SLC1A3 in different datasets. **(E)** Temporal expression changes of SLC1A3 in glioma progression. **(F)** Simplified GBmap annotation results based on the original annotation. **(G)** The expression level of SLC1A3 at the single-cell resolution. **(H)** The quantification of SLC1A3 in the different cell clusters. The unpaired Student’s t-test was used for P-value calculation. **(I)** Immunofluorescence results showed the presence of SLC1A3 in spontaneous glioma models and the localization of SLC1A3 with tumor cells (tumor in green and SLC1A3 in red, scale bar = 200μm). The unpaired Student’s t-test was used for P-value calculation. *P<0.05, **P<0.01, ***P<0.001, ****P<0.0001; ns, P >0.05.

To delineate the master regulators orchestrating the complex oncogenic trajectory observed in our single-cell models, and to identify therapeutically actionable targets at the tumor-stroma interface, we established a rigorous, integrative multi-omics filtering pipeline (**Figure 4A**). Recognizing that the profound microenvironmental crosstalk and synaptic alterations identified earlier may be mediated by membrane-associated elements, we intersected our bulk transcriptomic DEGs and aberrant AS events with the temporally dynamic drift genes derived from our pseudotime trajectory. To specifically capture the surfaceome reprogramming, this consensus gene list was further cross-referenced against a curated, comprehensive database of human cell-surface proteins. Through this stringent multidimensional selection, followed by survival-based filtering, we pinpointed exactly three membrane-associated candidates whose elevated expression was significantly and robustly correlated with adverse clinical outcomes in glioma patients: CLU, ADORA1, and SLC1A3 (**Figure 4A**). While CLU and ADORA1 have been previously documented in the context of tumor-immune microenvironment modulation and immunotherapy resistance (27–29), SLC1A3 emerged as a particularly compelling candidate. SLC1A3 encodes the excitatory amino acid transporter 1 (EAAT1), a canonical glutamate transporter fundamentally recognized for clearing extracellular glutamate and maintaining neural, synaptic, and metabolic homeostasis in the healthy central nervous system (30, 31). The spontaneous emergence of this specific transporter perfectly aligns with our prior GSEA and single-cell findings, mechanistically hinting that emerging glioma cells might actively hijack glutamate transport networks to fuel their aggressive phenotypes.

To definitively establish the temporal and spatial specificity of these candidates during early gliomagenesis, we turned to the subventricular zone (SVZ), the widely acknowledged neurogenic niche and putative anatomical epicenter of glioma origin (32). We systematically evaluated the expression profiles of these three candidates across paired human SVZ and matched mature tumor tissues. Strikingly, *SLC1A3* was the singular candidate that exhibited a profound, tumor-specific upregulation in public clinical cohorts (**Figure 4B**). To orthogonally validate this human in silico observation, we leveraged a spontaneous glioma mouse model, which faithfully recapitulates the natural developmental architecture of gliomagenesis (33). Consistent with the bioinformatic data, our *in vivo* transcriptomic quantification explicitly corroborated that *SLC1A3* expression is drastically amplified in the neoplastic core relative to the anatomically matched, non-malignant SVZ region (**Figure 4C**). We also found that IDH1 wild-type patients exhibited higher SLC1A3 expression at the transcriptional level compared with IDH1-mutant patients. The clinical gravity of this upregulation was further substantiated by a comprehensive Kaplan-Meier survival analysis across multiple independent, large-scale datasets. Across all cohorts, elevated SLC1A3 expression stratified patients into cohorts with markedly shorter overall survival, confirming its status as a robust prognostic determinant (**Figure 4D**). Furthermore, when we mapped SLC1A3 expression back onto our continuous single-cell pseudotime trajectory, we observed a striking temporal dynamic: rather than being constitutively active, SLC1A3 expression was progressively induced in a time-dependent manner as the precursor cells advanced toward full malignancy (**Figure 4E**). This suggests that SLC1A3 is not merely a byproduct of gliomagenesis, but rather a driving feature whose accumulation dictates the evolutionary trajectory of the tumor.

Having established the clinical and temporal significance of SLC1A3, we sought to resolve its precise cellular compartmentalization. We queried the comprehensive GBmap single-cell atlas, mapping SLC1A3 across a highly diverse landscape of malignant and stromal populations (**Figures 4F, G**) (34). UMAP clustering and quantitative analysis revealed that while SLC1A3 was detectable in tumor-associated macrophages (TAMs), its expression was overwhelmingly dominated by and concentrated within the malignant compartment (**Figure 4H**). Finally, to visualize this spatial hierarchy at the protein level, we performed immunofluorescence on tissue sections derived from our spontaneous glioma mouse model. The *in situ* architectural landscape unequivocally demonstrated dramatic enrichment of SLC1A3 within the tumor area, displaying extensive and near-exclusive co-localization with malignant tumor markers (**Figure 4I**). Collectively, this exhaustive multi-tier validation firmly establishes the membrane transporter SLC1A3 as a critical, clinically targetable hub governing the evolution of glioma-initiating cells.

### 2.5 SLC1A3 regulates the microenvironment disturbance of glioma

**Figure 5:**
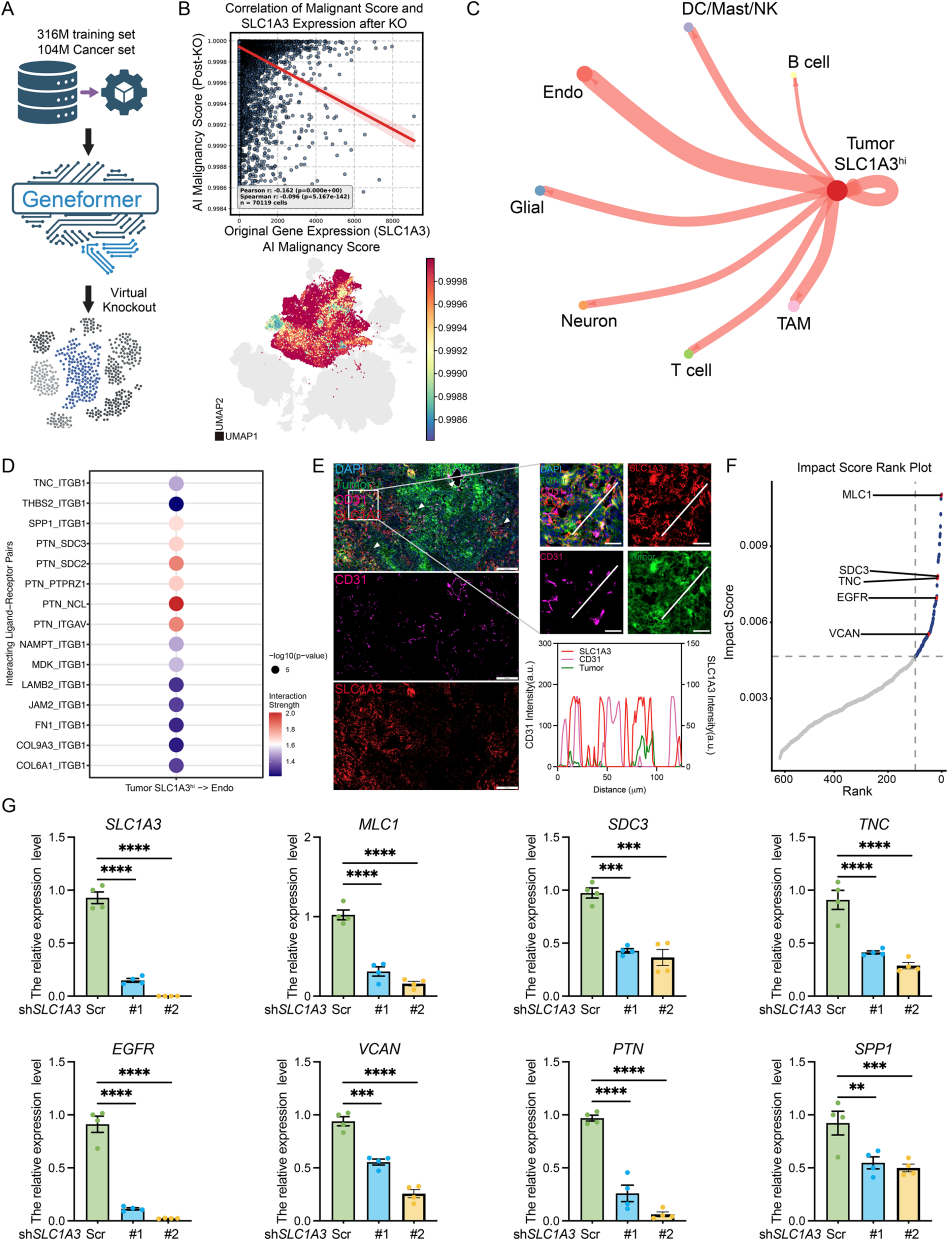
Cell interaction analysis inferred that SLC1A3^hi^ tumor cells affected the vascular niche function of glioma. **(A)** The workflow of virtual knockout. **(B)** The correlation of original *SLC1A3* expression with post-KO malignant score (upper) and the distribution of post-KO malignancy score based on AI calculation in tumor cells (bottom). **(C)** The CellChat analysis reveals the interaction strength between SLC1A3^hi^ tumor cells and other cells in TME. **(D)** The inferred interaction between SLC1A3^hi^ tumor cells and endothelial cells. **(E)** Immunofluorescence results showed SLC1A3^+^ tumor cells preferentially reside adjacent to the vasculature and spatial fluorescence quantitative results showed the distribution of SLC1A3 and CD31 (white line standing by the spatial fluorescence trajectory line, arrow indicates the SLC1A3^hi^ tumor cell nearby the vascular niche, scale bar = 200μm in the original figure and scale bar = 20μm in the zoom-in figure, tumor in green, CD31 in pink, and SLC1A3 in red). **(F)** The rank of gene changes after *SLC1A3* perturbation is shown, with the top 100 genes truncated in the dashed line and genes associated with vessels highlighted in red. **(G)** The quantification of the expression level of vessel associated gene and *SLC1A3* in U251MG following *SLC1A3* knockdown. The unpaired Student’s t-test was used for P-value calculation. **P<0.01, ***P<0.001, ****P<0.0001.

Having established the prognostic and developmental significance of SLC1A3, we next sought to mechanically define the dependencies associated with SLC1A3. To achieve a systems-level understanding of SLC1A3 dependency, without the inherent noise and metabolic artifacts of *in vitro* culture, we leveraged Geneformer (18), a cutting-edge foundation model pre-trained on a massive corpus of 104 million cancer single-cell transcriptomes (**Figure 5A**). By projecting our data into this context-aware embedding space, we executed an in silico zero-shot virtual knockout of SLC1A3 within the tumor. Strikingly, this targeted perturbation revealed profound transcriptomic fragility, precipitating a catastrophic collapse in the AI-inferred global malignancy score (**Figure 5B**). This confirms SLC1A3 is an indispensable hub for maintaining the intrinsic malignant state. Given our prior observation of extensive microenvironmental remodeling, we hypothesized that the malignant topology sustained by SLC1A3 might be physically coupled to specific niche interactions. Unbiased CellChat profiling revealed a highly polarized interactome, wherein SLC1A3^hi^ tumor cells engaged predominantly with vascular endothelial cells via canonical angiogenic and ECM cascades (**Figures 5C, D**). To robustly validate this spatial tropism *in situ*, we performed immunofluorescence on glioma tissues. Confirming the CellChat topology, SLC1A3^+^ tumor cells structurally organized themselves along the abluminal surface of CD31^+^ niche (**Figure 5E**). This physically anchors the SLC1A3^hi^ population within the perivascular niche (PVN), a critical microenvironment known to sustain glioma stemness and drive invasive progression.

To trace the downstream genetic networks driving this vascular crosstalk, we returned to Geneformer, utilizing a broader 316 million cell dataset to globally rank genes impacted by a secondary SLC1A3 perturbation (**Figure 5F**). Crucially, the top destabilized genes (MLC1, SDC3, TNC, EGFR, and VCAN) prominently featured TNC and SDC3, perfectly overlapping with the precise perivascular ligand-receptor pairs independently identified by our CellChat analysis. To empirically substantiate these profound bioinformatic and AI-derived predictions, we engineered U251MG human glioma cells with lentiviral shRNA to stably abrogate *SLC1A3* expression. Consistent with the in silico model, silencing *SLC1A3 in vitro* triggered a dramatic, highly significant downregulation of the entire predicted perivascular interactome (**Figure 5G**). Meanwhile, to further verify the results of virtual knockout, we performed cell counting in the U251MG cell line after knocking down SLC1A3, and the results confirmed that SLC1A3 knockdown could inhibit the proliferation of U251MG cells to a certain extent. Collectively, this multi-tier validation unequivocally defines SLC1A3 as a master regulator that couples the intrinsic malignant state of glioma cells with their ability to construct and hijack the perivascular niche.

**Figure 6:**
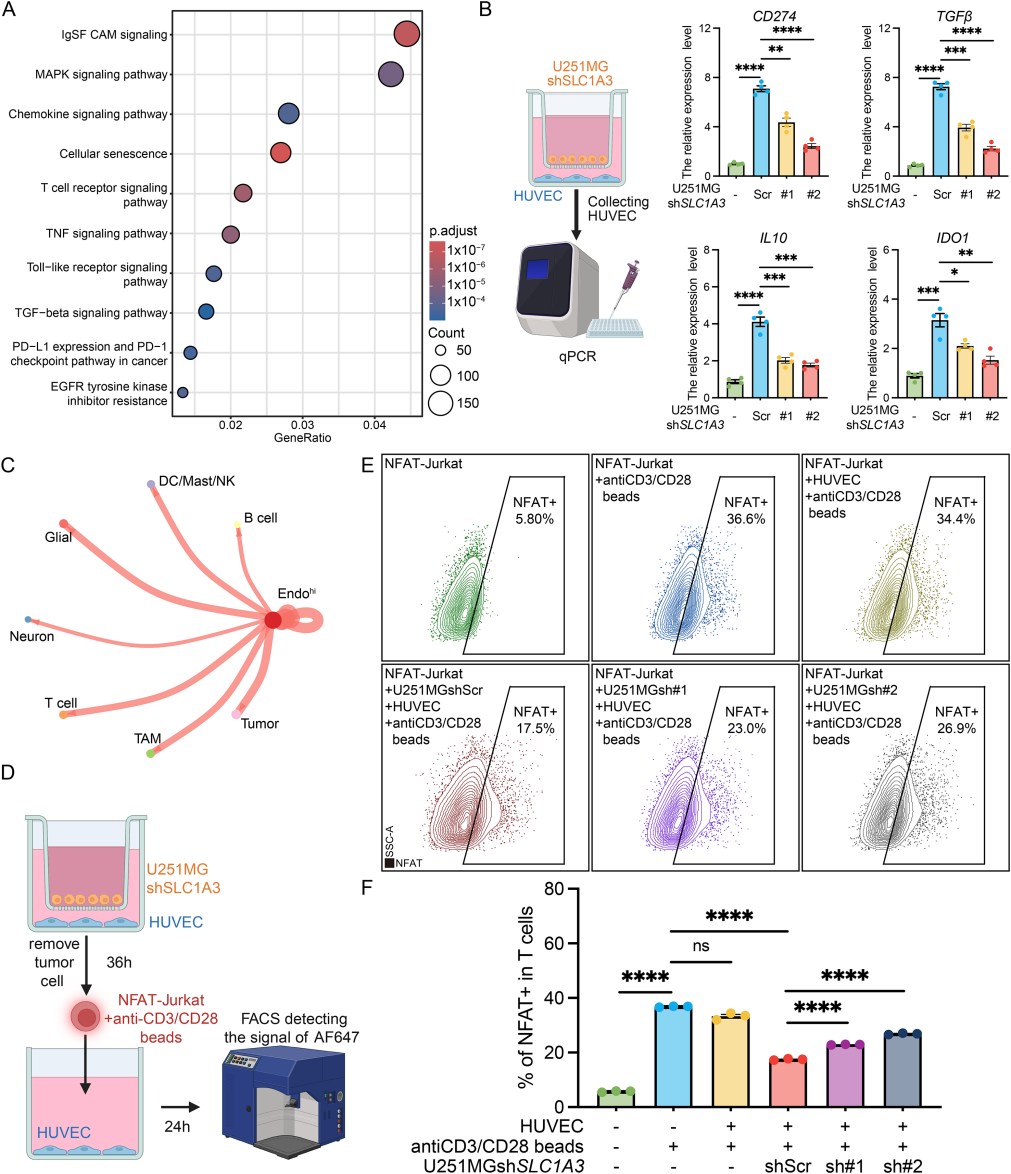
SLC1A3^hi^ tumor cells influence T-cell activation through the vascular niche. **(A)** GO enrichment results of shared upregulated genes in specific endothelial cells interacting with SLC1A3^hi^ tumor cells. **(B)** Co-culture system revealed that the SLC1A3 modulates endothelial cell-mediated immunosuppression. **(C)** The CellChat analysis reveals the interaction strength between specific endothelial cells interacting with SLC1A3^hi^ tumor cells (Endo^hi^) and other cells in TME. **(D)** The workflow of U251MG-HUVEC-T cell co-culture. **(E)** The flow cytometry after co-culture detects the activation level of Jurkat after co-culture. **(F)** The quantification of the activation level of Jurkat after co-culture. The unpaired Student’s t-test was used for P-value calculation. *P<0.05, **P<0.01, ***P<0.001, ****P<0.0001; ns, P >0.05.

Finally, to delineate the downstream functional consequences of this SLC1A3-driven perivascular niche, we sought to uncover how this interaction remodels the local immune microenvironment. Transcriptomic enrichment analysis of the specific endothelial subpopulation interacting with SLC1A3^hi^ tumor cells revealed a profound skewing toward immunomodulatory, senescence, and cytokine signaling cascades (**Figure 6A**). This suggested that the endothelium might act as a proxy to suppress local immunity. To model this *in vitro*, we co-cultured human umbilical vein endothelial cells (HUVECs) with U251MG glioma cells. Strikingly, glioma co-culture potently modifies the HUVECs, triggering a robust upregulation of canonical immunosuppressive mediators, including *CD274*, *TGFβ*, *IL10*, and *IDO1* (**Figure 6B**). Crucially, targeted knockdown of *SLC1A3* in the upstream tumor compartment substantially abrogated this paracrine induction, confirming that SLC1A3 dictates this endothelial reprogramming. These findings were further corroborated by spatial transcriptomic profiling of clinical samples (35), which demonstrated a robust upregulation of these immunosuppressive genes in tumor-associated endothelial cells (TECs) relative to normal-like endothelial cells (NECs). Together, these data indicate that the tumor microenvironment actively reprograms the vascular endothelium toward an immunosuppressive phenotype. But it might not be the only factor; it’s just that the SLC1A3 phenotype might be related to it.

Corroborating this *in vitro* finding, our extended CellChat interactome identified this highly reprogrammed endothelial cluster (Endo^hi^) as a central signaling hub projecting directly to the T cell compartment (**Figure 6C**). We therefore hypothesized that SLC1A3^hi^ glioma cells exploit the perivascular endothelium to orchestrate a trans-cellular immunosuppressive relay. Spatial feature mapping revealed a pronounced regional overlap between malignant SLC1A3 abundance and T cell exhaustion signatures. Spatial trajectory analyses extending from the SLC1A3-dense tumor core to the margin demonstrated that the dynamic decline of the T cell exhaustion score closely paralleled the spatial reduction of malignant SLC1A3. To rigorously quantify this proximity-dependent relationship, we calculated the minimum physical spatial distance between malignant spots and exhausted T cell niches. Notably, SLC1A3-high malignant cells were situated significantly closer to exhausted T cells than their SLC1A3-low counterparts (Wilcoxon rank-sum test, P < 0.001). Bivariate Moran’s I and Local Indicators of Spatial Association (LISA) analyses provided definitive statistical validation (36), identifying a highly significant positive spatial co-aggregation (Moran’s I > 0, P < 0.001) predominantly characterized by SLC1A3^hi^/Exhaustion^hi^ domains. To empirically test this tripartite interaction, we developed a sequential co-culture assay (**Figure 6D**). We pre-conditioned HUVECs with either WT or *SLC1A3*-knockdown tumor cells, removed the tumor cells, and subsequently introduced anti-CD3/CD28 beads-stimulated NFAT-Jurkat reporter T cells to the conditioned endothelium. Flow cytometric quantification of NFAT-driven fluorescence revealed a dramatic dynamic (**Figures 6E, F**), while HUVECs unstimulated by tumor cells exerted negligible effects on T cell activity, tumor-conditioned HUVECs profoundly blunted T cell activation. Most remarkably, genetic ablation of SLC1A3 in the primary tumor cells effectively broke this relay, significantly rescuing downstream T cell effector function. Collectively, these data uncover a novel mechanism whereby glioma cells leverage SLC1A3 to establish a highly immunosuppressive perivascular barrier, shielding the tumor from T cell-mediated clearance.

## 3 Discussion

The relentless progression and profound therapeutic resistance of GBM are fundamentally rooted in the evolutionary plasticity of GICs. While the phenotypic endpoints of GICs-mediated immune evasion are increasingly recognized, the dynamic ontogeny, specifically how corrupted NSCs programs cascade into multicellular immunosuppressive networks, has remained a critical blind spot. In this study, we provide a multiomics signature map of early gliomagenesis. By integrating transcriptomics, AS, and AI-driven single-cell foundation models, we identify SLC1A3 not merely as a prognostic biomarker, but as a master electro-metabolic hub that orchestrates a profound tumor-endothelial-immune trans-cellular relay, ultimately establishing an immune-privileged perivascular sanctuary.

A defining hallmark of our findings is the striking acquisition of neural and synaptic signatures during the NSCs-to-GICs transition. This aligns seamlessly with the emerging paradigm of cancer neuroscience (37, 38), which posits that malignant gliomas physically and electrochemically integrate into host neural circuits (39) to drive proliferation (e.g., neuron-glioma synapses) (40, 41). In this hyperactive neuro-oncological niche, neurotransmitters, specifically glutamate, act as potent mitogens driving tumor proliferation (42, 43). Strikingly, our stringent multi-omics pipeline pinpointed SLC1A3 (encoding the canonical inward glutamate transporter EAAT1) as a critical surface dependency. Previous studies have suggested that glutamate plays a significant role in maintaining the stemness of glioma, while our research fills the functional gap of SLC1A3 from tumor initiation to tumor stage (44). Rather than purely mediating extracellular neurotransmitter clearance, we propose that in the development of glioma, GICs actively hijack SLC1A3 to voraciously scavenge synaptic glutamate. Once internalized, glutamate serves as an indispensable carbon and nitrogen reservoir, fueling TCA cycle anaplerosis, nucleotide biosynthesis, and redox homeostasis. Thus, SLC1A3 acts as the crucial electro-metabolic bridge, allowing glioma cells to convert ambient neural circuit activity directly into aggressive oncogenic anabolism.

While our data establish SLC1A3 primarily as a malignant cell-enriched feature in glioblastoma, its detectable expression in TAMs warrants consideration. Macrophage activation states are inherently coupled to metabolic rewiring, with glutamine and glutamate metabolism playing pivotal roles in myeloid cell plasticity (45, 46). As a major glutamate and aspartate transporter, SLC1A3 on TAMs may facilitate the intracellular accumulation of these metabolites to fuel the tricarboxylic acid cycle and support redox homeostasis (30, 47). Such metabolic adaptations are typically required to sustain a pro-tumoral, anti-inflammatory phenotype (36). Consequently, SLC1A3 expression within the myeloid compartment may function cooperatively with malignant cell-derived SLC1A3 to deplete essential microenvironmental nutrients, thereby reinforcing the metabolic barriers that drive T cell exhaustion. Investigating its contribution to macrophage polarization remains a critical area for future research.

Crucially, our study pioneers the integration of large-scale AI foundation models (Geneformer) to transition from descriptive transcriptomics to predictive systems biology. Traditional differential expression analysis inherently fails to distinguish between correlative passenger genes and indispensable network drivers. By projecting our single-cell data into a 104-million-cell context-aware embedding space, our in silico zero-shot virtual knockout circumvented the metabolic artifacts of traditional *in vitro* models. The catastrophic collapse of the AI-inferred global malignancy score following SLC1A3 perturbation unequivocally defined its status as a keystone vulnerability. This application of AI highlights a paradigm shift in target discovery: leveraging global transcriptomic fragility to uncover targets that maintain the structural integrity of the malignant state. Beyond intrinsic tumor autonomous functions, our interactome analysis unravelled a striking spatial tropism: SLC1A3^hi^ tumor cells physically anchor themselves to the abluminal surface of neovasculature. The PVN is widely recognized as a critical microenvironment for maintaining GIC stemness (48); however, our study redefines the PVN as a dynamic immunosuppressive proxy. We discovered that SLC1A3^hi^ tumor cells secrete canonical ECM remodeling and angiogenic ligands (e.g., TNC, PTN) to potently “educate” the local endothelium.

Most remarkably, our sequential multicellular co-culture assays explicitly demonstrated that glioma cells do not necessarily suppress T cells directly; rather, they orchestrate a tripartite trans-cellular relay. The SLC1A3-educated endothelium undergoes a profound phenotypic shift, upregulating an arsenal of immunosuppressive mediators (*PD-L1*, *TGFβ*, *IL10*, and *IDO1*) that effectively paralyze infiltrating T cells. It has been observed in peripheral tumors that tumor cells with high expression of SLC1A3 can interact with fibroblasts and thereby inhibit the infiltration and activation of T cells (49). This mechanism partly explains why traditional immune checkpoint blockades (e.g., anti-PD-1) have overwhelmingly failed in GBM trials: the therapeutic antibodies cannot overcome the impenetrable, functionally corrupted vascular barrier (50, 51). By demonstrating that genetic ablation of SLC1A3 in the tumor compartment can dismantle this endothelial immunosuppressive programming and restore downstream T cell effector function, we unveil a novel strategy to “defrost” the immunologically cold glioblastoma microenvironment.

Beyond the direct crosstalk between malignant cells and the endothelium, the spatial architecture of the GBM perivascular niche is fundamentally characterized by severe hypoxic gradients (52). Such oxygen deprivation is a well-established driver of endothelial dysfunction, metabolic reprogramming, and widespread alternative splicing events that collectively promote a hostile TME (53–55). Given our findings on the SLC1A3-dependent immunosuppressive axis, it is highly probable that hypoxia acts synergistically with this metabolic vulnerability. Hypoxia-inducible factors are known to independently upregulate immunosuppressive mediators, including CD274 and IDO1 in TECs (56–58). Therefore, SLC1A3-driven amino acid/glutamate compartmentalization and hypoxia-induced endothelial dysregulation likely operate cooperatively. This dual mechanism would establish a profound physical and metabolic barrier within the perivascular niche, thereby exacerbating the exclusion and localized exhaustion of infiltrating T cells. Future studies delineating the mechanistic intersection between hypoxic signaling, alternative splicing, and SLC1A3-mediated metabolic rewiring will be crucial for comprehensively understanding vascular-mediated immunosuppression in GBM.

Despite these compelling findings, our study acknowledges certain limitations. While we mapped the downstream consequences of SLC1A3 on the vascular niche, the precise intracellular signaling cascades linking glutamate transport to the secretion of angiogenic ligands (e.g., TNC) warrant further mechanistic elucidation, potentially involving metabolic-epigenetic crosstalk. Future *in vivo* studies utilizing syngeneic models with functional immune systems will be crucial to evaluate whether pharmacological inhibition of SLC1A3 can synergize with existing immunotherapies. Translating this therapeutic vulnerability into clinical settings will require overcoming the blood-brain barrier. Current pharmacological strategies focus on selective allosteric inhibitors, such as UCPH-101 and UCPH-102, which may necessitate advanced nanocarrier delivery systems to achieve adequate intracranial efficacy (31, 59). Alternatively, precision modalities like antisense oligonucleotides or peptide-based vaccines offer high target specificity with minimized systemic toxicity (60–62). Pairing these targeted SLC1A3 interventions with immune checkpoint blockade presents a rational clinical strategy to dismantle the metabolic barriers of the vascular niche and reprogram the “cold” TME.

In conclusion, our integrative study decodes the evolutionary trajectory of glioma and defines SLC1A3 as a critical nexus coupling intrinsic malignant stability with microenvironmental subversion. By hijacking the perivascular niche to establish an immunosuppressive endothelial barrier, SLC1A3 shields the tumor from immunological clearance. Targeting this specific tumor-endothelial-T cell axis offers a highly promising therapeutic vulnerability, potentially reprogramming the glioblastoma ecosystem to restore anti-tumor immunity.

## 4 Materials and methods

### 4.1 Cell line culture

HEK293T (from ATCC), DF1 (from ATCC), and U251MG (from ATCC) were cultured in DMEM with 10% FBS and 1x penicillin/streptomycin. NFAT-Jurkat was cultured in RPMI1640 with 10% FBS and 1x penicillin/streptomycin. HEK293T, U251MG, and NFAT-Jurkat were grown at 37 °C with 5% CO2. DF1 was grown at 39 °C with 5% CO2.

### 4.2 Lentivirus preparation and infection

Two shRNAs targeting human SLC1A3 (target sequence: 5’-CGACAGTGAAACCAAGATGTA-3’ and 5’-CCGACCATACAGAATGAGCTA-3’) and a non-targeting control shRNA (shScr, 5’-CCTAAGGTTAAGTCGCCCTCG-3’) were cloned into the PLKO.1., psPAX2 (Addgene, #12260), and pMD2.G (Addgene, #12259) with PEI (Yeasen, #40815ES) co-transfected into HEK293T to produce lentiviral particles. Viruses were collected after transfection and precipitated by PEG8000 (Sigma, #89510). For lentiviral infection, cells were incubated with viral particles for 24 h, followed by 0.5μg/ml puromycin to select infected cells to obtain stable cells. Stable cells were verified by RT-qPCR.

### 4.3 Co-culture assay

U251MG cells and HUVECs were co-cultured at a 1:1 ratio using a Transwell system, with HUVECs seeded in the lower chamber and U251MG cells in the upper chamber. After 36 h of co-culture, tumor cells in the upper chamber were removed, and the medium was replaced with a 1:1 mixture of NFAT-Jurkat medium and tumor-conditioned medium. NFAT-Jurkat cells were then added at an NFAT-Jurkat-to-HUVEC ratio of 3:1, together with anti-CD3/CD28 beads (1×10^6^ cells per 25 µL; STEMCELL Technologies, #10971). After a further 24 h of co-culture, the supernatant was collected for subsequent flow cytometric analysis.

### 4.4 qRT-PCR

Total cellular RNA was isolated using RNA purification kit (EZB, #B0004DP), and the tissue RNA was isolated using tissue RNA purification kit (EZB, # EZB-RN001-plus). And reverse transcribed using 4X EZscript Reverse Transcription Mix II (EZB, #RT2GQ). qRT-PCR was performed on a QuantStudio 6 Flex Real-Time PCR System (Thermo Fisher Scientific) using 2X Color SYBR Green qPCR Master Mix (EZB, #A0012-R2).

### 4.5 Construction of RCAS mouse models

N-tva mice are obtained from the Jackson Laboratory. All mouse experiments followed an animal protocol approved by the Institutional Animal Care and Use Committee of the Shanghai Medical College, Fudan University. The animals were sacrificed at the Day21 for the tissue RNA isolated and sectioned, following the protocol outlined by the NIH Office of Animal Care and Use. Brain tissues were harvested, fixed in 4% formaldehyde, dehydrated in 30% sucrose, embedded in OCT (Sakura, #4583), and sectioned for frozen analysis. These sections were stored at -80 °C.

DF1 cells were expanded to passage 4 and transfected with RCAS-PDGF-HA and RCAS-shTrp53 (33) using a Fugene 6 Transfection kit (Roche, #11814443001). Transfected DF1 cells were used for injections after 7 days. DF1 cells were injected as equal mixtures of 10^5^ cells/uL, 1 μL per mouse, using a 30-gauge needle attached to a Hamilton syringe.

### 4.6 Flow cytometry

The co-cultured cell NFAT-Jurkat was harvested from the suspension (the NFAT-Jurkat is a suspension cell line). After harvesting, the cells were washed with SWB buffer (2% FBS in PBS) 3 times at 4 °C. Then, cells were stained with BD Horizon™ Fixable Viability Stain 780 (BD, #565388) at 4 °C for 30 min in the dark to discriminate live or dead cells. Then cells were washed with SWB buffer 3 times at 4 °C and suspended in 200μL SWB and detected using APC-Cy7 and Alexa Fluor 647 channels.

### 4.7 Immunofluorescence staining of tissue

For immunofluorescence staining, the sections were blocked for 2h at RT, and primary antibodies were incubated at 4 °C overnight (CD31 1:300, R&D Systems, AF3628; HA 1:300, CST, #2367; SLC1A3, 1:300, Proteintech, 20785-1-AP). The secondary antibodies were incubated for one hour incubated with Alexa Flour™ 594 goat anti-rabbit IgG (Invitrogen, A-11012), Alexa Flour™ 488 goat anti-mouse IgG (Invitrogen, A-11001), and Alexa Flour™ 647 donkey anti-goat IgG (Invitrogen, A-21447) for 1 hour, after primary antibody incubation and PBS wash. The results were captured using the Olympus SLIDEVIEW VS200.

### 4.8 RNA-seq data analysis

The downloaded raw data were aligned to the hg38 reference genome by STAR (v2.7.11b) for further analysis. For differential expression analysis, the R package DESeq2 (v1.50.2) was used to analyze the differences in NSCs and GICs and the differential genes in P < 0.05 and |LogFC| > 1.2. For KEGG and GO enrichment, clusterProfiler (v4.18.4) was used for enrichment analysis, and ggplot2 (v4.0.2) for plotting. For the alternative splicing analysis, we used the rMATS (v4.3.0) and followed the protocol of Xing et al. (63). For survival analysis, survival (v3.8-6) and survminer (v0.5.2) were used.

### 4.9 scRNA-seq data analysis

For the scRNA-seq, we followed the analysis procedure provided by Kim et al. (26). In brief, we downloaded their dataset and followed the code provided in the article to perform stepwise analysis and obtain fastMNN integration data alongside their original annotation results. Then, we isolated the Pre-CC and tumor clusters for reintegration and made pseudotime analysis using the R package slingshot (v2.18.0). Then, for the CellChat, we use FastCCC (64) to calculate all 338,564 cells in GBmap based on the original annotation. And the virtual knockout was using Geneformer V2 (18) for the malignant score in the 104M_CLcancer model and the 316M model for whole cell perturbation. All computational inferences are utilized on a dedicated NVIDIA A800 PCIe Tensor Core GPU (80 GB VRAM) and 512 GB of system RAM. The computational pipeline was implemented in Python (v3.10), leveraging the PyTorch deep learning framework (v2.0.1, with CUDA 11.8 integration). Given the massive memory requirements for extracting 1,024-dimensional gene-level embeddings (using the “cls_and_gene” embedding mode) across thousands of target cells, the inference process was highly optimized to prevent out-of-memory (OOM) failures. Specifically, the high-expressing target cell datasets were dynamically partitioned into manageable chunks (chunk_size = 800 cells), and the forward pass was strictly constrained to a forward_batch_size = 1. Explicit memory management and garbage collection (gc.collect(), torch.cuda.empty_cache()) were enforced between chunks to maintain GPU VRAM stability throughout the full-transcriptome in silico perturbation analysis. Plots were generated by Seurat (v5.4.0).

### 4.10 Spatial transcriptomics analysis

Spatial transcriptomics data of human GBM were processed using the SPATA2 package (v3.1.4). To overcome the multi-cellular resolution limitation of Visium ST spots and precisely define the malignant compartment, we applied the SpaCET (v1.2.0) deconvolution algorithm to estimate the proportion of malignant and endothelial cells within each spot. To eliminate stromal artifacts, a “Pure Malignant SLC1A3 Score” was calculated by weighting the raw SLC1A3 expression by the deconvoluted malignant cell fraction. Subsequently, to quantify the spatial relationship between SLC1A3^+^ malignant cells and immune infiltration, spatial spots were categorized based on their Pure Malignant SLC1A3 score and CD8A expression (or T cell Exhaustion Score). To statistically determine the spatial exclusion, the Bivariate Moran’s I statistic was calculated utilizing the spdep package (v1.4). A spatial weight matrix was constructed based on a 6-nearest neighbor (k=6) network, and the significance of the spatial association was robustly tested using 999 Monte Carlo permutations.

Furthermore, to investigate proximity-driven microenvironmental changes, we calculated the minimum Euclidean distance from each spot to the SLC1A3^+^ malignant core. The local microenvironment was stratified into concentric annular zones extending outward to evaluate spatial gradients of T cell exhaustion. Dynamic spatial trajectories from the tumor core to the margin were constructed, and smoothed expression profiles were fitted using LOESS to visualize the spatial segregation of SLC1A3 and exhausted T cells. Finally, to validate the *in vitro* endothelial findings *in vivo*, a multi-patient ST cohort analysis was performed. Vascular-enriched spots (top 50% SpaCET-derived endothelial fraction) were stratified into TEC areas and NEC areas based on the co-existing malignant cell fraction (top 30% vs. bottom 30%, respectively). Intra-patient comparisons of the mean expression of immunosuppressive mediators (CD274, TGFB1, IL10, and IDO1) between matched TECs and NECs were statistically evaluated using the Paired Wilcoxon rank-sum test. All visualizations and statistical analyses were performed in R utilizing ggplot2 (v4.0.2) and ggpubr (v0.6.3).

### 4.11 Quantitative and statistical analysis

R (v4.5) and Python (v3.11.6) were used for all bioinformatics analysis. Prism software (version 10) was also used. Survival curves were statistically analyzed using the Log-rank test and plotted using Kaplan-Meier curves. For the rest of the data, unpaired, two-sided Student’s t tests were used, and considered P<0.05 to be significant, specifying *P<0.05, **P<0.01, ***P<0.001, ****P<0.0001, ns, P >0.05.

## Data Availability

The original contributions presented in the study are included in the article/**Supplementary Material**. Further inquiries can be directed to the corresponding authors.

## References

[1] Wu J Heidelberg RE Gajjar A . Adolescents and young adults with cancer: Cns tumors. J Clin Oncol. (2024) 42:686–95. doi: 10.1200/jco.23.01747. PMID: 38064656 PMC11550794

[2] Weller M Wen PY Chang SM Dirven L Lim M Monje M . Glioma. Nat Rev Dis Primers. (2024) 10:33. doi: 10.1038/s41572-024-00516-y. PMID: 38724526

[3] Sanai N Berger MS . Surgical oncology for gliomas: The state of the art. Nat Rev Clin Oncol. (2018) 15:112–25. doi: 10.1038/nrclinonc.2017.171. PMID: 29158591

[4] Gimple RC Bhargava S Dixit D Rich JN . Glioblastoma stem cells: Lessons from the tumor hierarchy in a lethal cancer. Genes Dev. (2019) 33:591–609. doi: 10.1101/gad.324301.119. PMID: 31160393 PMC6546059

[5] Singh SK Hawkins C Clarke ID Squire JA Bayani J Hide T . Identification of human brain tumour initiating cells. Nature. (2004) 432:396–401. doi: 10.1038/nature03128. PMID: 15549107

[6] Couturier CP Ayyadhury S Le PU Nadaf J Monlong J Riva G . Single-cell rna-seq reveals that glioblastoma recapitulates a normal neurodevelopmental hierarchy. Nat Commun. (2020) 11:3406. doi: 10.1038/s41467-020-17186-5. PMID: 32641768 PMC7343844

[7] Jeon HM Kim SH Jin X Park JB Kim SH Joshi K . Crosstalk between glioma-initiating cells and endothelial cells drives tumor progression. Cancer Res. (2014) 74:4482–92. doi: 10.1158/0008-5472.Can-13-1597. PMID: 24962027 PMC4295931

[8] Li D Cui G Yang K Lu C Jiang Y Zhang L . Inhibiting macrophage-derived lactate transport restores cgas-sting signalling and enhances antitumour immunity in glioblastoma. Nat Cell Biol. (2026) 28:349–62. doi: 10.1038/s41556-025-01839-y. PMID: 41495200

[9] Yuan H Wu X Wu Q Chatoff A Megill E Gao J . Lysine catabolism reprograms tumour immunity through histone crotonylation. Nature. (2023) 617:818–26. doi: 10.1038/s41586-023-06061-0. PMID: 37198486 PMC11089809

[10] Barthel FP Johnson KC Varn FS Moskalik AD Tanner G Kocakavuk E . Longitudinal molecular trajectories of diffuse glioma in adults. Nature. (2019) 576:112–20. doi: 10.1038/s41586-019-1775-1. PMID: 31748746 PMC6897368

[11] Suvà ML Tirosh I . The glioma stem cell model in the era of single-cell genomics. Cancer Cell. (2020) 37:630–6. doi: 10.1016/j.ccell.2020.04.001. PMID: 32396858

[12] PiaoW LeeZL ZapasG WuL JewellCM AbdiR . Regulatory t cell and endothelial cell crosstalk. Nat Rev Immunol. (2025) 25:588–607. doi: 10.1038/s41577-025-01149-2. PMID: 40169744

[13] Hua Y Vella G Rambow F Allen E Antoranz Martinez A Duhamel M . Cancer immunotherapies transition endothelial cells into HEVs that generate Tcf1(+) T lymphocyte niches through a feed-forward loop. Cancer Cell. (2022) 40 (12):1600–18.e10. doi: 10.1016/j.ccell.2022.11.002. PMID: 36423635 PMC9899876

[14] Slansky JE Spellman PT . Alternative splicing in tumors - a path to immunogenicity? N Engl J Med. (2019) 380:877–80. doi: 10.1056/NEJMcibr1814237. PMID: 30811916

[15] Venkataramany AS Schieffer KM Lee K Cottrell CE Wang PY Mardis ER . Alternative rna splicing defects in pediatric cancers: New insights in tumorigenesis and potential therapeutic vulnerabilities. Ann Oncol. (2022) 33:578–92. doi: 10.1016/j.annonc.2022.03.011. PMID: 35339647 PMC12361925

[16] Zhang Y Qian J Gu C Yang Y . Alternative splicing and cancer: A systematic review. Signal Transduct Target Ther. (2021) 6:78. doi: 10.1038/s41392-021-00486-7. PMID: 33623018 PMC7902610

[17] Su J Song Y Zhu Z Huang X Fan J Qiao J . Cell-cell communication: New insights and clinical implications. Signal Transduct Target Ther. (2024) 9:196. doi: 10.1038/s41392-024-01888-z. PMID: 39107318 PMC11382761

[18] Theodoris CV Xiao L Chopra A Chaffin MD Al Sayed ZR Hill MC . Transfer learning enables predictions in network biology. Nature. (2023) 618:616–24. doi: 10.1038/s41586-023-06139-9. PMID: 37258680 PMC10949956

[19] Cui H Wang C Maan H Pang K Luo F Duan N . Scgpt: Toward building a foundation model for single-cell multi-omics using generative ai. Nat Methods. (2024) 21:1470–80. doi: 10.1038/s41592-024-02201-0. PMID: 38409223

[20] Yates J Van Allen EM . New horizons at the interface of artificial intelligence and translational cancer research. Cancer Cell. (2025) 43:708–27. doi: 10.1016/j.ccell.2025.03.018. PMID: 40233719 PMC12007700

[21] Bose A Rhrissorrakrai K Utro F Parida L . Advancing single-cell omics and cell-based therapeutics with quantum computing. Nat Rev Mol Cell Biol. (2026). doi: 10.1038/s41580-025-00918-0. PMID: 41478876

[22] Fischer DS Villanueva MA Winter PS Shalek AK . Adapting systems biology to address the complexity of human disease in the single-cell era. Nat Rev Genet. (2025) 26:514–31. doi: 10.1038/s41576-025-00821-6. PMID: 40065155 PMC13552703

[23] Constantinou M Nicholson J Zhang X Maniati E Lucchini S Rosser G . Lineage specification in glioblastoma is regulated by mettl7b. Cell Rep. (2024) 43:114309. doi: 10.1016/j.celrep.2024.114309. PMID: 38848215 PMC11220825

[24] Vinel C Rosser G Guglielmi L Constantinou M Pomella N Zhang X . Comparative epigenetic analysis of tumour initiating cells and syngeneic epsc-derived neural stem cells in glioblastoma. Nat Commun. (2021) 12:6130. doi: 10.1038/s41467-021-26297-6. PMID: 34675201 PMC8531305

[25] Wang Y Xie Z Kutschera E Adams JI Kadash-Edmondson KE Xing Y . Rmats-turbo: An efficient and flexible computational tool for alternative splicing analysis of large-scale rna-seq data. Nat Protoc. (2024) 19:1083–104. doi: 10.1038/s41596-023-00944-2. PMID: 38396040

[26] Kim HJ Kim KW Cha DH Yoo J Kim EH Chang JH . Precancerous cells initiate glioblastoma evolution and contribute to intratumoral heterogeneity. Cancer Discov. (2025) 15:1377–91. doi: 10.1158/2159-8290.Cd-24-0234. PMID: 40233712

[27] Ren X Chang C Qi T Yang P Wang Y Zhou X . Clusterin is a prognostic biomarker of lower-grade gliomas and is associated with immune cell infiltration. Int J Mol Sci. (2023) 24(17):13413. doi: 10.3390/ijms241713413. PMID: 37686218 PMC10487477

[28] Li HJ Yu ZY Gao HP Xu YR Li XY Jiang W . Inhibiting adora1 enhances glioma apoptosis and increases its sensitivity to anti-pd1 therapy. Front Oncol. (2025) 15:1545780. doi: 10.3389/fonc.2025.1545780. PMID: 40376586 PMC12078947

[29] Huang J Chen MN Du J Liu H He YJ Li GL . Differential expression of adenosine p1 receptor adora1 and adora2a associated with glioma development and tumor-associated epilepsy. Neurochem Res. (2016) 41:1774–83. doi: 10.1007/s11064-016-1893-1. PMID: 27038930

[30] Tajan M Hock AK Blagih J Robertson NA Labuschagne CF Kruiswijk F . A role for p53 in the adaptation to glutamine starvation through the expression of slc1a3. Cell Metab. (2018) 28:721–736.e6. doi: 10.1016/j.cmet.2018.07.005. PMID: 30122553 PMC6224545

[31] Canul-Tec JC Assal R Cirri E Legrand P Brier S Chamot-Rooke J . Structure and allosteric inhibition of excitatory amino acid transporter 1. Nature. (2017) 544:446–51. doi: 10.1038/nature22064. PMID: 28424515 PMC5410168

[32] Lee JH Lee JE Kahng JY Kim SH Park JS Yoon SJ . Human glioblastoma arises from subventricular zone cells with low-level driver mutations. Nature. (2018) 560:243–7. doi: 10.1038/s41586-018-0389-3. PMID: 30069053

[33] Yang J Yang H Yuan Y Zhang C Fu Z Chen Y . Prickle4 drives microenvironmental remodeling and resistance to parp inhibition in idh-mutant glioma. Adv Sci (Weinh). (2026) 13:e03866. doi: 10.1002/advs.202503866. PMID: 41236163 PMC12806547

[34] Ruiz-Moreno C Salas SM Samuelsson E Minaeva M Ibarra I Grillo M . Charting the single-cell and spatial landscape of idh-wild-type glioblastoma with gbmap. Neuro Oncol. (2025) 27:2281–95. doi: 10.1093/neuonc/noaf113. PMID: 40312969 PMC12526130

[35] Ravi VM Will P Kueckelhaus J Sun N Joseph K Salié H . Spatially resolved multi-omics deciphers bidirectional tumor-host interdependence in glioblastoma. Cancer Cell. (2022) 40:639–655.e13. doi: 10.1016/j.ccell.2022.05.009. PMID: 35700707

[36] Wang L Chu H Chen D Wei Y Jia J Li L . Slc2a1(+) tumour-associated macrophages spatially control cd8(+) t cell function and drive resistance to immunotherapy in non-small-cell lung cancer. Nat Cell Biol. (2026) 28:338–48. doi: 10.1038/s41556-025-01840-5. PMID: 41501177 PMC12904792

[37] Zhang S Yuan L Lin P Yang G Zhou X Xu J . Cancer neuroscience: Signaling pathways and new therapeutic strategies for cancer. Signal Transduct Target Ther. (2026) 11(1):66. doi: 10.1038/s41392-025-02364-y. PMID: 41724757 PMC12926231

[38] Mancusi R Monje M . The neuroscience of cancer. Nature. (2023) 618:467–79. doi: 10.1038/s41586-023-05968-y. PMID: 37316719 PMC11146751

[39] Venkatesh HS Morishita W Geraghty AC Silverbush D Gillespie SM Arzt M . Electrical and synaptic integration of glioma into neural circuits. Nature. (2019) 573:539–45. doi: 10.1038/s41586-019-1563-y. PMID: 31534222 PMC7038898

[40] Barron T Yalçın B Su M Byun YG Gavish A Shamardani K . Gabaergic neuron-to-glioma synapses in diffuse midline gliomas. Nature. (2025) 639:1060–8. doi: 10.1038/s41586-024-08579-3. PMID: 39972132 PMC11946904

[41] Drexler R Drinnenberg A Gavish A Yalçin B Shamardani K Rogers AE . Cholinergic neuronal activity promotes diffuse midline glioma growth through muscarinic signaling. Cell. (2025) 188(17):4640–57.e30. doi: 10.1016/j.cell.2025.05.031. PMID: 40541184 PMC12396346

[42] Taylor KR Barron T Hui A Spitzer A Yalçin B Ivec AE . Glioma synapses recruit mechanisms of adaptive plasticity. Nature. (2023) 623:366–74. doi: 10.1038/s41586-023-06678-1. PMID: 37914930 PMC10632140

[43] Takano T Lin JH Arcuino G Gao Q Yang J Nedergaard M . Glutamate release promotes growth of Malignant gliomas. Nat Med. (2001) 7:1010–5. doi: 10.1038/nm0901-1010. PMID: 11533703

[44] Restall IJ Cseh O Richards LM Pugh TJ Luchman HA Weiss S . Brain tumor stem cell dependence on glutaminase reveals a metabolic vulnerability through the amino acid deprivation response pathway. Cancer Res. (2020) 80:5478–90. doi: 10.1158/0008-5472.Can-19-3923. PMID: 33106333

[45] Delgado ER Patel P Tao J Krutsenko Y Liu S Green D . Glutamine synthetase loss in β-catenin-mutant hepatocellular carcinoma promotes tumor burden through macrophage metabolic reprogramming. Hepatology. (2025). doi: 10.1097/hep.0000000000001591. PMID: 41129335

[46] Muri J Kopf M . Redox regulation of immunometabolism. Nat Rev Immunol. (2021) 21:363–81. doi: 10.1038/s41577-020-00478-8. PMID: 33340021

[47] Choi J Stradmann-Bellinghausen B Yakubov E Savaskan NE Régnier-Vigouroux A . Glioblastoma cells induce differential glutamatergic gene expressions in human tumor-associated microglia/macrophages and monocyte-derived macrophages. Cancer Biol Ther. (2015) 16:1205–13. doi: 10.1080/15384047.2015.1056406. PMID: 26047211 PMC4623498

[48] Adjei-Sowah EA O'Connor SA Veldhuizen J Lo Cascio C Plaisier C Mehta S . Investigating the interactions of glioma stem cells in the perivascular niche at single-cell resolution using a microfluidic tumor microenvironment model. Adv Sci (Weinh). (2022) 9:e2201436. doi: 10.1002/advs.202201436. PMID: 35619544 PMC9313491

[49] Xiang S Chen Y Wang C Wang M He Y Liu Z . Alleviated t cell exhaustion and slc1a3-mediated stroma-remodelling dictate chemoimmunotherapy efficacy in oesophageal squamous cell carcinoma. Gut. (2026) 75:252–64. doi: 10.1136/gutjnl-2025-335642. PMID: 40983502

[50] van Tellingen O Yetkin-Arik B de Gooijer MC Wesseling P Wurdinger T de Vries HE . Overcoming the blood-brain tumor barrier for effective glioblastoma treatment. Drug Resist Update. (2015) 19:1–12. doi: 10.1016/j.drup.2015.02.002. PMID: 25791797

[51] Gilbertson RJ Rich JN . Making a tumour's bed: Glioblastoma stem cells and the vascular niche. Nat Rev Cancer. (2007) 7:733–6. doi: 10.1038/nrc2246. PMID: 17882276

[52] Wang W Li T Cheng Y Li F Qi S Mao M . Identification of hypoxic macrophages in glioblastoma with therapeutic potential for vasculature normalization. Cancer Cell. (2024) 42:815–832.e12. doi: 10.1016/j.ccell.2024.03.013. PMID: 38640932

[53] Rankin EB Giaccia AJ . Hypoxic control of metastasis. Science. (2016) 352:175–80. doi: 10.1126/science.aaf4405. PMID: 27124451 PMC4898055

[54] Wei X Chen Y Jiang X Peng M Liu Y Mo Y . Mechanisms of vasculogenic mimicry in hypoxic tumor microenvironments. Mol Cancer. (2021) 20:7. doi: 10.1186/s12943-020-01288-1. PMID: 33397409 PMC7784348

[55] LaGory EL Giaccia AJ . The ever-expanding role of Hif in tumour and stromal biology. Nat Cell Biol. (2016) 18:356–65. doi: 10.1038/ncb3330. PMID: 27027486 PMC4898054

[56] Ding XC Wang LL Zhang XD Xu JL Li PF Liang H . The relationship between expression of Pd-L1 and Hif-1α in glioma cells under hypoxia. J Hematol Oncol. (2021) 14:92. doi: 10.1186/s13045-021-01102-5. PMID: 34118979 PMC8199387

[57] Shen SC Dey S DuHadaway JB Sutanto-Ward E Hampton MT Kozlov SV . Neovascular pruning by Ido1 inhibitors can potentiate immunogenic cytotoxicity of ischemia-targeted agents to synergistically enhance anti-Pd-1 responsiveness. J Immunother Cancer. (2025) 13(5):e011398. doi: 10.1136/jitc-2024-011398. PMID: 40447318 PMC12128478

[58] Chen J Jiang CC Jin L Zhang XD . Regulation of Pd-L1: A novel role of pro-survival signalling in cancer. Ann Oncol. (2016) 27:409–16. doi: 10.1093/annonc/mdv615. PMID: 26681673

[59] Jiang H Zhang N Tang T Feng F Sun H Qu W . Target the human alanine/serine/cysteine transporter 2(Asct2): Achievement and future for novel cancer therapy. Pharmacol Res. (2020) 158:104844. doi: 10.1016/j.phrs.2020.104844. PMID: 32438035

[60] Khuu A Verreault M Colin P Tran H Idbaih A . Clinical applications of antisense oligonucleotides in cancer: A focus on glioblastoma. Cells. (2024) 13(22):1869. doi: 10.3390/cells13221869. PMID: 39594617 PMC11592788

[61] Mahdi J Trivedi V Monje M . The promise of immunotherapy for central nervous system tumours. Nat Rev Immunol. (2026) 26:213–29. doi: 10.1038/s41577-025-01227-5. PMID: 41053233

[62] Weller M Roth P Preusser M Wick W Reardon DA Platten M . Vaccine-based immunotherapeutic approaches to gliomas and beyond. Nat Rev Neurol. (2017) 13:363–74. doi: 10.1038/nrneurol.2017.64. PMID: 28497804

[63] Shen S Park JW Lu Z Lin L Henry MD Wu YN . Rmats: Robust and flexible detection of differential alternative splicing from replicate Rna-Seq data. Proc Natl Acad Sci. (2014) 111:E5593–E601. doi: 10.1073/pnas.1419161111. PMID: 25480548 PMC4280593

[64] Hou S Ma W Zhou X . Fastccc: A permutation-free framework for scalable, robust, and reference-based cell-cell communication analysis in single cell transcriptomics studies. Nat Commun. (2025) 16:11428. doi: 10.1038/s41467-025-66272-z. PMID: 41390348 PMC12749929

