# Supplementary figures and images for: Glioma-intrinsic SLC1A3 hijacks the vascular niche to establish an immunosuppressive microenvironment
Source: Front Immunol. 2026 Apr 23;17:1824726. doi: 10.3389/fimmu.2026.1824726 (PMC13149450; doi:10.3389/fimmu.2026.1824726)

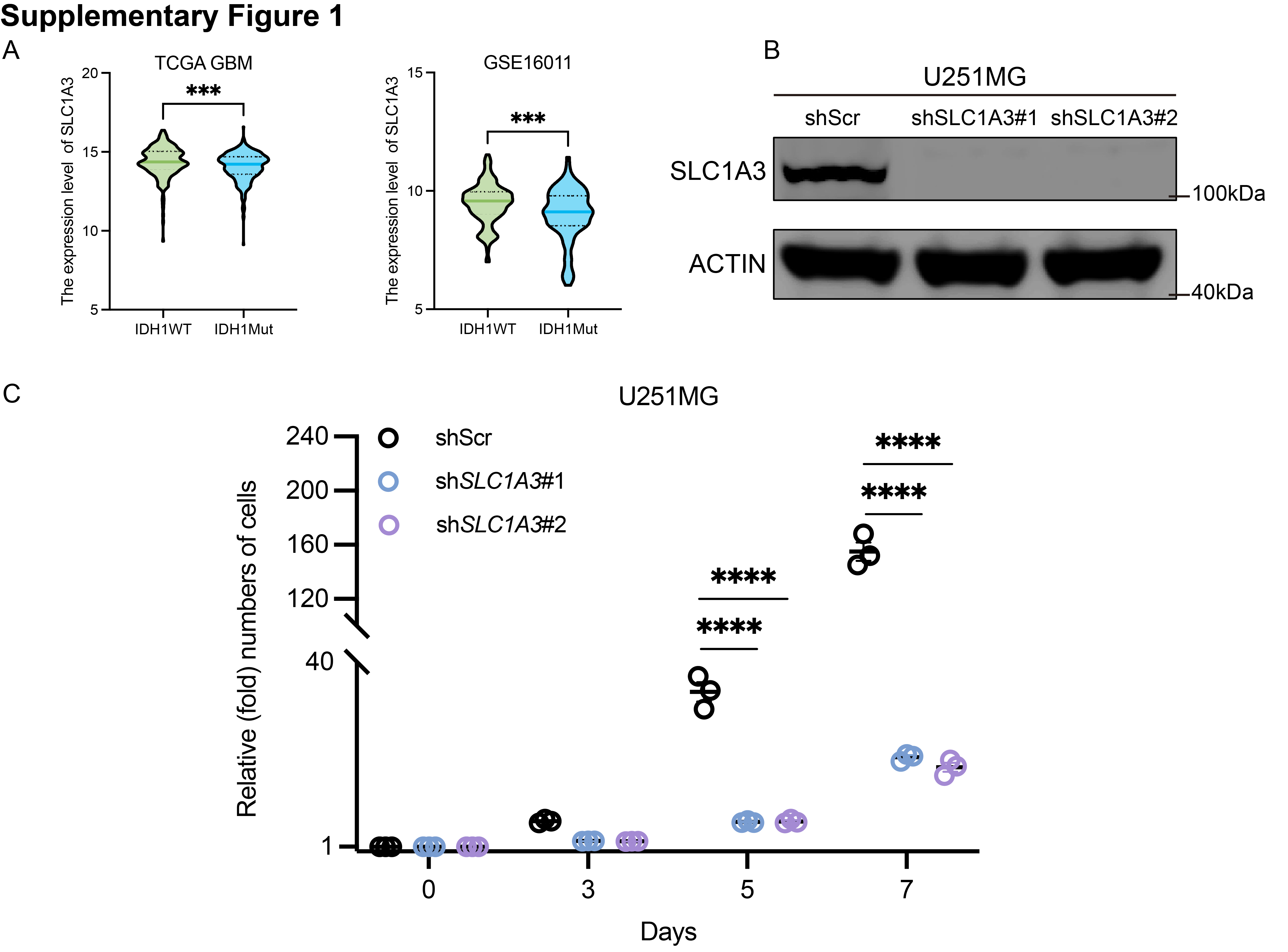

Supplement: Supplementary file 1 [file Image1.tif]

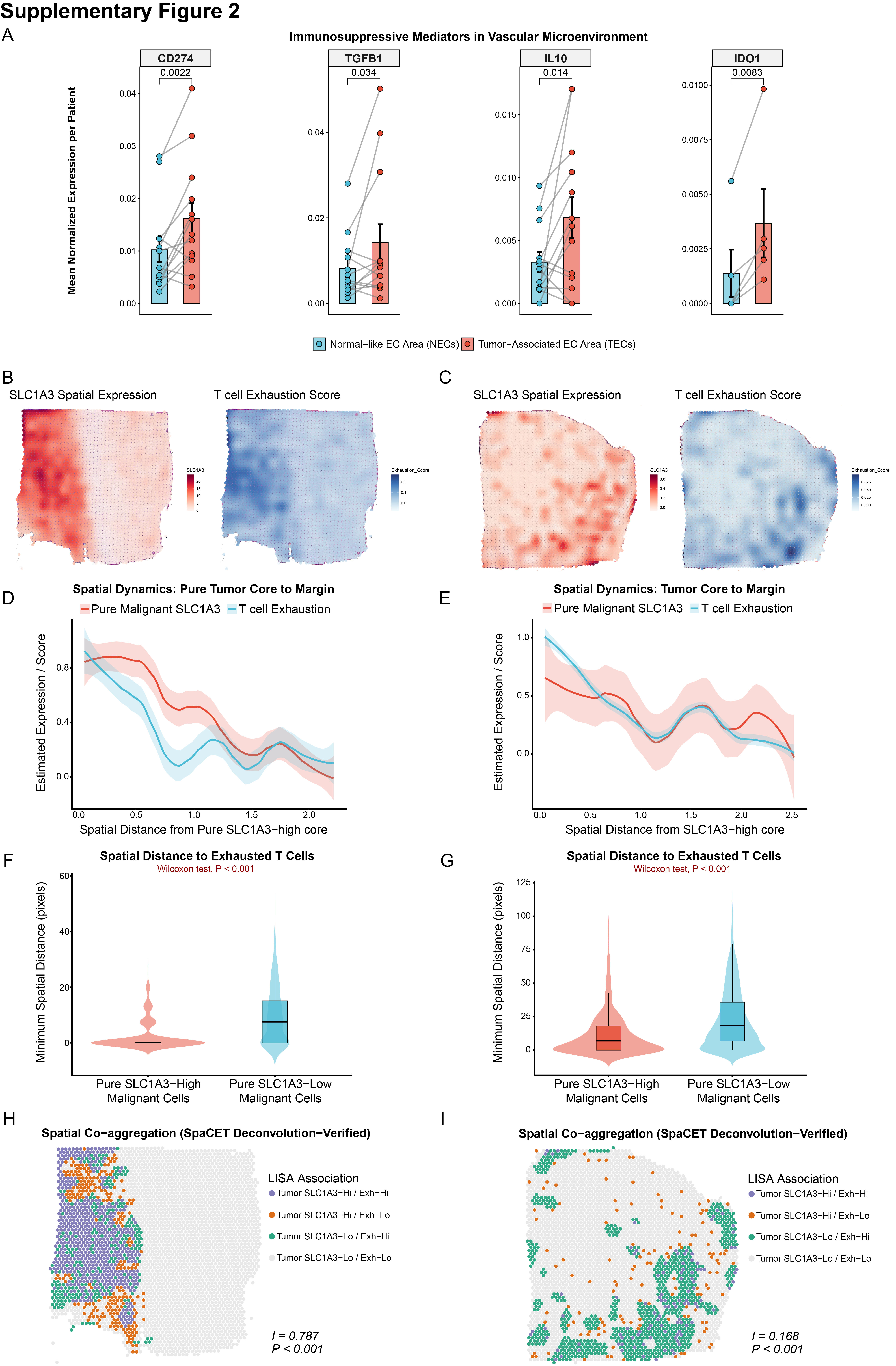

Supplement: Supplementary file 2 [file Image2.tif]
